# Context Emotionality and Second Language Proficiency Influence Vocabulary Acquisition: An ERP Study

**DOI:** 10.3390/bs16060925

**Published:** 2026-06-05

**Authors:** Shunhua Liu, Yutong Li

**Affiliations:** 1 School of Education Science, Anshun University, Anshun 561000, China; liushunhua2328@asu.edu.cn; 2 School of Psychology, Liaoning Normal University, Dalian 116029, China

**Keywords:** context emotionality, second language proficiency, vocabulary acquisition, ERP

## Abstract

To investigate the neural mechanisms underlying how context emotionality and second language (L2) proficiency influence novel word acquisition, we recruited 48 college students and asked them to learn new words within emotional contexts. We recorded the event-related potentials (ERPs) during word recognition and selected the N1 (70–160 ms) component at frontal and central sites and the late positive complex (LPC; 500–750 ms) component at frontal, central, and parietal sites for statistical analysis. The results showed that words learned in a positive context elicited larger N1 waves in the left frontal region and smaller LPC waves in frontal regions than words learned in a negative context. Furthermore, in the higher-proficiency group, words learned in a negative context induced larger LPC waves in the left posterior region than in the lower-proficiency group. These findings led us to three conclusions: (1) A positive context activates neural responses more rapidly and attenuates them during the late processing stage. (2) L2 fluency modulates the extent to which a negative context affects new word recognition. (3) Higher fluency amplifies the role of learning context in word processing. This study sheds light on how the emotional context and L2 proficiency jointly shape the neural dynamics of vocabulary learning, and future research should explore these mechanisms across diverse learner populations and with more naturalistic learning tasks to better inform emotion-integrated language instruction.

## 1. Introduction

### 1.1. The Facilitative Effect of Emotional Context on L2 Novel Word Acquisition

Vocabulary acquisition is an important stage of language learning, and it is significantly enhanced when learned in context. The semantic associations between novel words and their contexts promote memory retention (Twomey & Westermann, 2018). Novel words refer to words that learners have not come across before. Emotional contexts give words a certain emotional meaning (Guo et al., 2018). Emotional context refers to the sentential frame in which a novel word is embedded, carrying either positive or negative emotional valence. Understanding the emotional context can deepen the encoding of new words, enhance memory, and accelerate the transition from new exposure to long-term mastery. In second language (L2) acquisition, the influence of context emotionality on word acquisition has attracted much scholarly attention. Studies have suggested that emotional experiences of native language and L2 are different (Davydova et al., 2025; Marian & Kaushanskaya, 2004). Bilinguals perceive less emotional information in their L2 than in their native language (Champoux-Larsson & Nook, 2024; Dewaele, 2010). Even when bilinguals are aware of the semantic and emotional content of second language words, their emotional experience is often less vivid than that of native speakers (Del Maschio et al., 2024; Opitz & Degner, 2012). Frances et al. (2020) embedded positive and neutral pseudowords into positive and neutral contexts. Intermediate-level English learners whose native language was Spanish were asked to learn these pseudowords, and the influence of context emotionality on L2 word acquisition was investigated. The results showed that a positive context can promote novel word acquisition more effectively than neutral contexts. Driver (2022) introduced a negative context into their research and found that for native and Hispanic-origin foreign language learners, both neutral and negative contexts were more conducive to L2 vocabulary acquisition than positive contexts. Frances et al.’s research presented pseudowords in context, and the meaning of pseudowords was inferred based on the context. Positive contexts had a greater effect on positive new words than neutral contexts did on neutral new words. Subjects used by Driver (2022) were special, including native speakers and Hispanic foreign language learners. It was found that positive contexts had the poorest effect and mainly reflected the situation of native speakers. The two studies had different subjects, different experimental conditions, and did not isolate the emotionality of novel words when exploring the role of emotional context. To address this limitation, Snefjella et al. (2020) asked participants to study neutral pseudowords in English to explore the influence of emotional context on vocabulary acquisition. The results confirmed that emotional contexts are more conducive to vocabulary acquisition than neutral contexts. It can be seen that emotional contexts can independently affect L2 novel word acquisition.

### 1.2. L2 Proficiency Moderates the Effect of Emotional Context on L2 Novel Word Acquisition

Context, particularly emotional context, undeniably shapes how new words are learned. However, this shaping effect does not operate in isolation—it interacts with learner-specific variables, among which L2 proficiency serves as a key moderator. Specifically, learners with low L2 proficiency primarily rely on L1 vocabulary to achieve semantic activation of L2 words, whereas those with higher L2 proficiency develop a direct mapping between L2 forms and conceptual meaning (Lee & Schallert, 1997). This proficiency-driven shift is well captured by the competitive model (Hernandez et al., 2005), which posits that lexical access—the process of retrieving word information from the mental lexicon—gradually transitions from indirect (L1-mediated) to direct (L2-to-concept) access as proficiency increases. Given this theoretical framework, a critical question remains: does L2 proficiency moderate the influence of emotional context on novel word acquisition? While the role of emotion in vocabulary learning is well documented, no study to date has directly examined whether the emotional context produces distinct early (N1) and late (LPC) processing patterns during L2 novel word learning, or whether these patterns are shaped by proficiency level. This represents a major empirical gap that ERP methodology is uniquely positioned to fill. To address this gap, the present study employed a memory–recognition paradigm. Participants with high and low L2 proficiency memorized neutral new words embedded in positive, negative, and neutral contexts. During the recognition phase, both groups judged whether each word had been previously encountered while their brain electrical responses were recorded. Beyond its theoretical contributions, the discovery of contextual emotional dependence in vocabulary learning holds practical implications for instructional design and the quality of vocabulary teaching, while the demonstrated moderating effect of L2 proficiency provides empirical support for personalized teaching approaches.

### 1.3. Neural Indicators of Emotional Context’s Effects on L2 Word Acquisition

This study focused on the brain’s two electrical components: N1 and LPC. N1 is a negative wave that peaks around 100–200 ms after stimulus onset. The early selection model of attention holds that attention selection occurs in the early stage of sensory processing (Lavie, 1995). That is, the brain already begins to pay attention to the selection of stimuli during the early stage of sensory processing. Studies have found that emotional information induces a larger N1 amplitude in the frontal brain region compared to neutral information, indicating that emotional information is more likely to attract attention (Hamilton et al., 2014). In L2 word recognition tasks, the N1 amplitudes induced by positive words are larger than those induced by negative words, indicating that priority allocation of attention to emotional information also occurs in L2 contexts (Jończyk et al., 2024). The LPC (late positive component), generally peaking between 500 and 800 ms over centro-parietal sites, displays sustained elaborate evaluation of emotional contents (Huang et al., 2026). The LPC is highly sensitive to valence and subtle differences in emotional information. Increases in the LPC amplitude usually reflect a more detailed processing of emotional stimuli (Tang et al., 2026). Other studies have found that the frontal LPC components are related to the control process of emotional valence processing. Bayer et al. (2010) investigated the EEG responses to negative and neutral verbs in a semantic correctness judgment task and found that negative words elicited larger frontal LPC amplitudes than neutral words. The researchers suggested that emotional valence involves the evaluation process in the late processing stage. An increase in the frontal LPC amplitude indicates that the more evaluative processes emotional valence triggers, the more cognitive resources are consumed.

### 1.4. Study Aims and Hypotheses

Although existing studies have conducted multi-dimensional explorations of the relationship between emotional context and second language vocabulary acquisition, accumulating relatively rich empirical data, there are still several key issues that need to be further clarified and systematically verified. First, the conclusions of existing research on the effects of different valence sentiment contexts on the learning of new second language words are not yet unified, and the core influencing mechanism still needs to be further clarified. Secondly, the regulatory effect of second language proficiency on the processing of new second language words in emotional contexts has not been systematically explored at the neural mechanism level by existing research. Thirdly, there is still room for improvement in the fit between the experimental paradigms of existing research and the real vocabulary learning scenarios of second language learners. Therefore, this study takes Chinese English learners as the subjects and examines the influence of emotional context and second language proficiency on the learning of new second language words from the neural response level.

Firstly, this study hypothesizes that the emotional context affects L2 vocabulary acquisition. During the recognition stage, the contextual information from the learning stage is reactivated when the word is presented again. Correspondingly, it is expected that words learned in positive contexts will elicit larger N1 amplitudes and smaller LPC amplitudes. Secondly, the study predicts that the influence of emotional context on L2 vocabulary acquisition is regulated by L2 proficiency. The higher the fluency in a second language, the more deeply the context is processed, the greater the influence of emotional contex on the learning of new words. Finally, the study hypothesizes that the moderating effect of L2 proficiency is mainly reflected in the recognition of words learned in negative contexts; that is, individuals with higher L2 proficiency are expected to allocate more cognitive resources to recognize words learned in negative contexts than those with lower proficiency. This increased cognitive effort is anticipated to result in larger LPC amplitudes. That is, this research further explores the relationship between emotional context, L2 proficiency, and vocabulary acquisition.

## 2. Materials and Methods

### 2.1. Participants

According to Cohen (2013), an ideal statistical test requires both statistical power and an effect size that exceeds 0.8. Based on this standard, G*Power 3.1.9.7 software (http://www.gpower.hhu.de/, accessed on 1 May 2022) was used to calculate the sample size. G*Power is a free, open-source software designed for statistical power analysis and sample size planning across a wide range of tests. The experimental design was a 2 (group: higher proficiency, lower proficiency) × 3 (context valence: positive, negative, neutral) mixed factorial design. The parameters were set as follows: α = 0.05, f = 0.25, and 1 − β = 0.8. The resulting calculation indicated a required sample size of 28 participants. We posted information about recruiting subjects on the campus website. To ensure robustness, 48 college students were recruited. All participants were native Chinese speakers with English as their L2. The sample included 24 participants in the higher-proficiency group and 24 in the lower-proficiency group. The higher-proficiency group consisted of students who had passed CET-6 (20 females, mean age = 23.17 years). The lower-proficiency group included students who had not passed CET-4 (14 females, mean age = 22.08 years). All participants had normal or corrected-to-normal vision and were right-handed. Before the experiment, the participants provided informed consent. The participants were clear about the purpose of the experiment, the scope of data usage, the rights and obligations of participating in the experiment, withdrawal from the experiment, etc. They were compensated with 40 RMB after the experiment. This experiment was approved by the university’s Ethics Committee (LL2024235).

### 2.2. Stimuli and Procedure

First, 90 pseudowords were created, each composed of six letters. These pseudowords represented ten types of nouns, including food, musical instruments, furniture, etc. These nouns were neutral.

To generate 90 distractor words, one letter in each pseudoword was randomly replaced. For each pseudoword, a corresponding learning context was compiled, resulting in 90 contexts. Each context consisted of two sentences. The first sentence was 6 to 11 words long and carried an emotional valence determined by the emotional valence of the two words in the sentence, both sharing the same valence. The second sentence, 4 to 9 words long, was semantically related to the first sentence but neutral. To avoid bias, the second sentence used the third person as the subject. The second sentence included the pseudoword to be learned, with its meaning given in parentheses after the pseudoword (see Table 1). We recruited 20 college students to rate the valence of words and the first sentence on a 7-point scale, with 1 representing negative, 4 representing neutral, and 7 representing positive. The average negative score was below 1.5, the average neutral score was between 3.5 and 4.5, and the average positive score was above 6.5.

By substituting the two valence-determining words into the first sentence (see Table 1) of each context (fun–delicious, awful–smelly, quick–brown), three emotional categories were created: positive, negative, and neutral. A total of 270 contexts were divided into three lists. Each list contained 30 of each of the three contexts and were presented in a pseudo-random order. Each pseudoword was only learned in one emotional context, and each participant was exposed to only one list. The three lists were balanced in their presentation order through a Latin square sequence.

The experimental design was a 2 (group: higher proficiency, lower proficiency) × 3 (context valence: positive, negative, neutral) mixed factorial design. The experiment consisted of two stages: memory and recognition. The experimental program was implemented using E-Prime 3.0. During the learning stage, we informed the participants that they should read each sentence presented to them carefully and remember the new words in the second clause.

**Figure 1:**
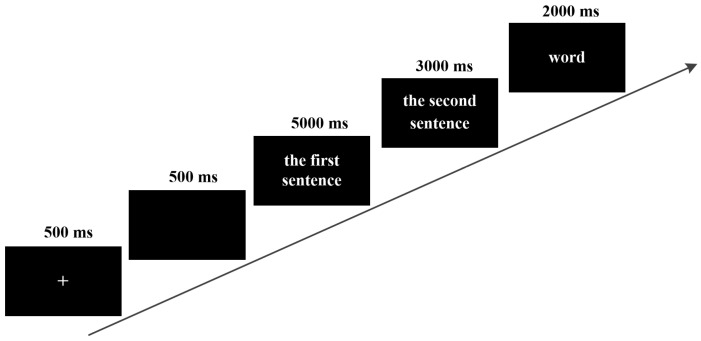
The flow diagram of the memory stage.

The trial flow for the memory stage is shown in Figure 1.

First, a fixation cross was presented in the center of the screen for 500 ms, followed by a blank screen for 500 ms. The first sentence with the context then appeared, lasting 5000 ms. The duration was based on the research by Altarriba and Basnight-Brown (2011), which suggests that enhances the emotional impact of the sentence and promotes vocabulary memory. Next, the second sentence for the that context was shown for 3000 ms. Finally, the learned pseudowords were presented independently for 2000 ms, and participants were asked to carefully memorize the words before the next trial.

Next, the participants wore electrode caps, sat in front of a computer, and entered the recognition stage. Press the “F” key for learned words or the “J” key.

**Figure 2:**
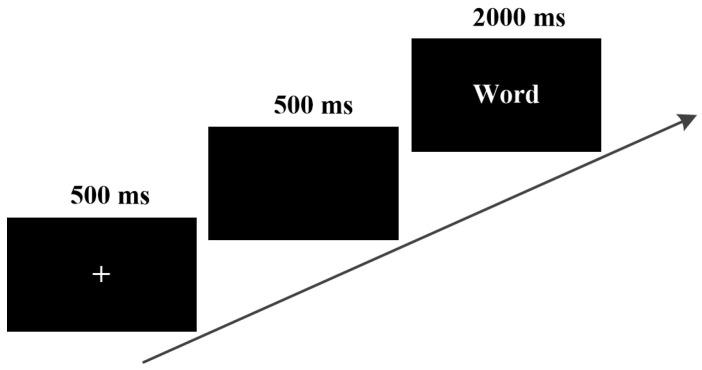
The recognition phase flow chart.

The process of the recognition trial is shown in Figure 2. The key assignment was balanced across participants. After a key press, the trial proceeded to the next word. If no response was made within 2000 ms, the trial proceeded. There were 90 pseudowords and 90 disturbing words, with 180 tries. The experiment recorded the participants’ electrical brain responses when the words were presented. In addition, before the formal experiment, the participants completed a set of practice trials to familiarize themselves with the experimental procedure.

### 2.3. EEG Recording

This study was approved by the Institutional Review Board of Liaoning Normal University (Approval No. LL2024235). All participants provided written informed consent prior to the EEG experiment. The EEG data were de-identified and stored under alphanumeric codes (e.g., P001, P002) rather than personal identifiers. The linking key between the codes and personal information was stored separately on an encrypted, access-controlled server. All the data were encrypted and accessible only to the authorized research team. In accordance with the Declaration of Helsinki (1964), the participants retained the right to withdraw at any time and request deletion of their data without penalty.

The electroencephalogram (EEG) data were collected using a 64-Channel Brain Products system. The electrode FPz served as the ground, and the electrode FCz was recorded for reference. The vertical electrooculogram (EOG) was recorded with an electrode placed below the right eye to monitor eye movements and blinks. All electrode impedances were kept below 5 kΩ, and the EEG signals were digitized online at 1000 Hz.

The raw EEG data were processed offline using Brain Vision Analyzer version 2.0 (Brain Products, GmbH; Gilching, Germany). The data were re-referenced to the average reference of left and right mastoids, then filtered with a digital band-pass of 0.1 and 30 Hz (slope 24 dB/oct) using an FIR filter. Ocular artifacts were corrected through an independent component analysis (ICA). The components identified as eye-related based on the frontal topography and blink-related time courses were excluded, and the remaining components were back-projected to the scalp channels. The ocular-corrected EEG data were segmented into epochs ranging from −200 ms before the stimulus to 800 ms after stimulus onset, with the 200 ms pre-stimulus period as the baseline. Additionally, epochs containing artifacts with peak-to-peak deflections exceeding ±80 μV were discarded. After artifact removal, the mean numbers of usable trials were as follows: 14.73 (SD = 0.47) for the highly proficient–positive condition; 14.23 (SD = 1.19) for the highly proficient–negative condition, and 14.77 (SD = 0.39) for the highly proficient–neutral condition. For the lower-proficiency group, the mean number of usable trials was 14.41 (SD = 0.70) for the positive condition; 14.27 (SD = 0.93) for the negative condition; and 14.23 (SD = 1.12) for the neutral condition. The resulting artifact-free data were averaged according to each trial type. Four subjects were excluded due to a large number of EEG artifacts. Finally, the data of 44 subjects were analyzed.

### 2.4. Data Analysis

**Figure 3:**
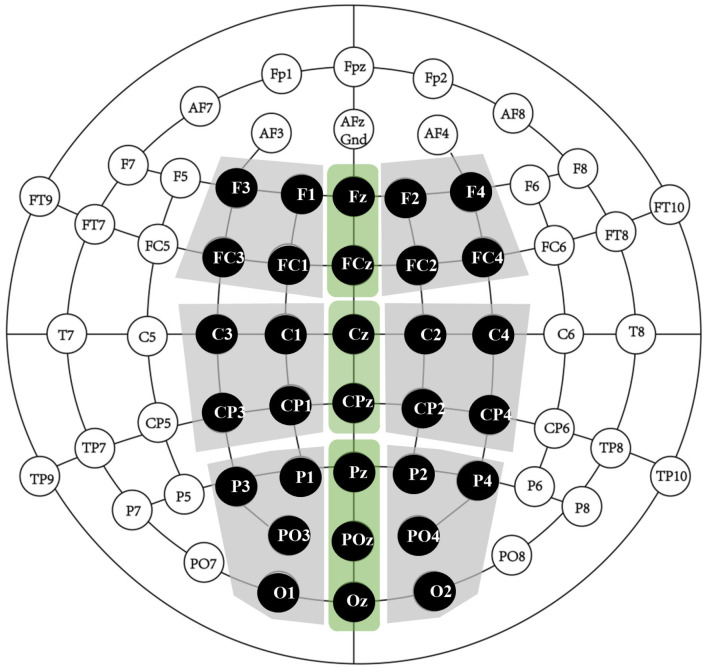
Topographical clusters and electrode positions. The green and gray areas demonstrate the midline cluster and the other six clusters.

The study specifically aimed to investigate the N1 and LPC components, with electrode selection tailored to each component. The regions of interest were chosen based on the previous literature (Hamilton et al., 2014; Schacht & Sommer, 2009; Bayer et al., 2010). The N1 (70–160 ms) component in frontal and central sites, and the LPC (500–750 ms) component in frontal, central and parietal sites were chosen for statistical analysis. The regions of interest are shown in Figure 3, including: left anterior (F1, F3, FC1 and FC3 electrodes), middle anterior (Fz, FCz), right anterior (F2, F4, FC2, FC4), left central (C1, C3, CP1, CP3), middle central (Cz and CPz), right central (C2, C4, CP2, CP4), left posterior (P1, P3, PO3, O1), middle central (Pz, POz, Oz) and right posterior (P2, P4, PO4, O2).

The amplitudes of N1 were analyzed using two 2 (group: higher proficiency, lower proficiency) × 3 (valence: positive, negative, neutral) × 3 (hemisphere: left, middle, right) mixed ANOVAs for the frontal and central sites. Moreover, three 2 × 3 × 3 mixed ANOVAs were performed on the frontal, central and posterior sites to analyze the amplitudes of the LPC component. In these analyses, the valence (positive, negative, neutral) and hemisphere (left, middle, right) were the within-subject factors, while the group (higher proficiency, lower proficiency) was the between-subject factor.

All statistical analyses reported *p*-values less than 0.05 as statistically significant. The Greenhouse–Geisser method was used to correct for degrees of freedom, and effect sizes were reported using partial eta-squared (η_p_^2^). Post hoc tests for significant main effects were conducted using the Tukey HSD approach, and simple tests involved the Bonferroni method. A Holm–Bonferroni correction was used to control for multiple comparisons across the ANOVAs.

## 3. Results

**Figure 4:**
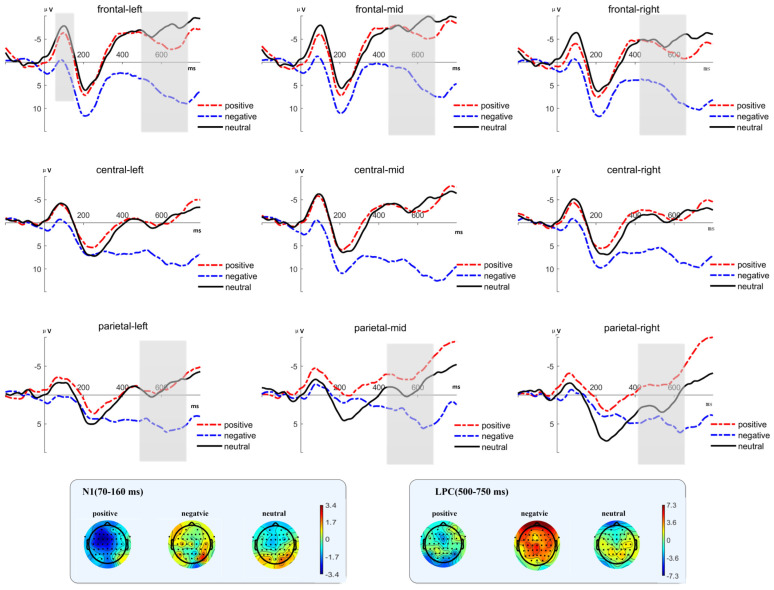
Waveforms and topographic maps of N1 and LPC components induced by words studied in different valence contexts.

The average amplitude graphs of electroencephalograms (EEGs), under different conditions, in different brain regions, and among different groups during the recognition stage, are shown in Figure 4. The first row shows the left, middle and right images of the frontal lobe brain region; the second row shows the left, middle and right images of the middle lobe brain region; and the third row shows the left, middle and right images of the parietal lobe brain region.

**N1 (70–160 ms)**

The time window of N1 was 70–160 ms. It can be seen from Figure 4 that the amplitude of N1 varied greatly. A three-factor repeated measures analysis of variance (ANOVA) of 2 (group) × 3 (context valence) × 3 (position: left, middle, right) was conducted on the average amplitude of N1 at different positions. The following only reports the significant main effects and interaction effects.

In the frontal sites, a significant main effect of group was observed—*F* (1, 42) = 5.91, *p* = 0.02, and η_p_^2^ = 0.12—suggesting a medium effect size (Cohen, 2013), meaning that approximately 12% of the variance in the dependent variable could can be attributed to this variable. The higher-proficiency group elicited larger N1 amplitudes than the lower-proficiency group. A significant interaction between valence and hemisphere was also found—*F* (4, 168) = 2.82, *p* = 0.03, and η_p_^2^ = 0.06—indicating a medium effect size. Further analyses revealed that the N1 amplitude for the negative condition was smaller than that for the positive condition in the left hemisphere—∆ = 3.51, 95% CI [0.01, 7.00], and *p* = 0.048. However, no significant differences in the N1 amplitudes were found between the valence conditions in the middle and right hemispheres, *p*s > 0.12. See Figure 4.

In the central sites, the main effects of group, *F* (1, 42) = 2.21 and *p* = 0.15, and valence, *F* (1.68, 70.39) = 1.74 and *p* = 0.19, were not significant. None of the interactions were significant, *p*s > 0.45.

**LPC (500–750 ms)**

A three-factor repeated measures analysis of variance (ANOVA) of 2 (group) × 3 (context valence) × 3 (position: left, middle, right) was conducted on the average amplitude of LPC at different positions.

**Figure 5:**
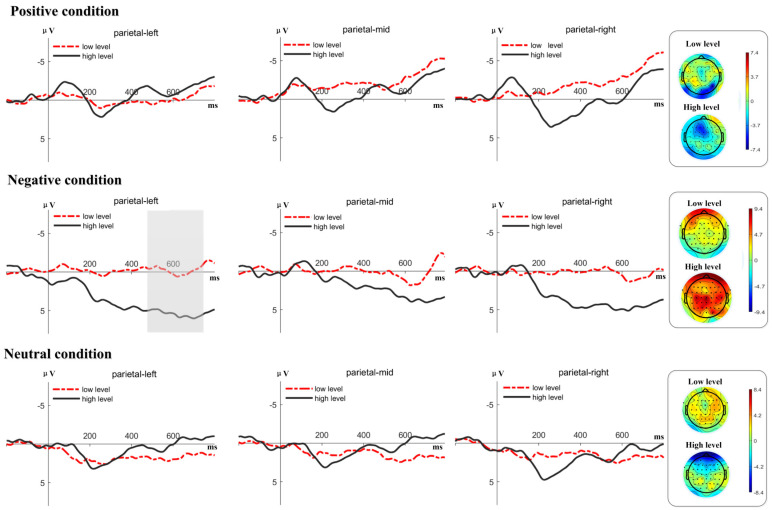
Waveforms and topographic maps of the LPC components induced by words studied in different groups under three valence contexts.

The results for the frontal sites revealed a significant main effect of valence—*F* (2, 84) = 3.87, *p* = 0.03, and η_p_^2^ = 0.03—suggesting a small effect size. The negative condition elicited larger LPC amplitudes than the positive condition, *p* = 0.03. However, the negative condition and the neutral condition were not significant, *p* = 0.11. No significant differences in the LPC amplitudes between the positive and neutral conditions were observed, *p* = 1.00. Moreover, a three-way interaction between valence, group and hemisphere was significant—*F* (4, 168) = 3.47, *p* = 0.01, and η_p_^2^ = 0.08—suggesting limited practical significance. However, further analyses found no differences between the groups across the hemisphere and valence conditions, *p*s > 0.17. See Figure 5.

In the central sites, a significant interaction between valence and hemisphere was observed—*F* (4, 168) = 3.38, *p* = 0.01, and η_p_^2^ = 0.07—suggesting a medium effect size. However, no significant differences between valence conditions in the left, middle, and right hemispheres were found, *p*s > 0.07.

The ANOVA results for the parietal site showed a significant main effect of valence— *F* (1.75, 73.39) = 3.78, *p* = 0.03, and η_p_^2^ = 0.08—suggesting a medium effect size. No significant differences between conditions were observed, *p*s > 0.07. In addition, a three-way interaction between valence, group, and hemisphere was found—*F* (4, 168) = 3.22, *p* = 0.01, and η_p_^2^ = 0.07—indicating a medium effect size. For the negative condition, the higher-proficiency group elicited larger LPC amplitudes than the lower-proficiency group in the left hemisphere—∆ = 5.69, 95% CI [0.75, 10.64], and *p* = 0.03. The differences between groups across the other hemispheres and valence conditions were not significant, *p*s > 0.07.

## 4. Discussion

This study mainly investigated the neural mechanism behind the influence of emotional context and L2 proficiency on novel word acquisition during English vocabulary learning. A word memory–recognition paradigm was used, with neutral pseudowords (new words) first learned in different emotional contexts during the memory stage, followed by an investigation into EEG differences between the two groups during the recognition stage when these words were presented again. The results showed that the emotional context influences vocabulary acquisition. Words learned in a positive context elicited larger N1 amplitudes and smaller LPC amplitudes in the left frontal region compared to words learned in a negative context. L2 proficiency affected vocabulary acquisition. The higher-proficiency group produced a larger N1 amplitude in the frontal brain region than the lower-proficiency group. It is worth noting that the influence of emotional context on new word acquisition was also regulated by L2 proficiency. For the words learned in a negative context, the higher-proficiency group showed larger LPC amplitudes than the lower-proficiency group in the left posterior brain region.

In this experiment, bilinguals were asked to memorize new words in context, where context played a dual role. On the one hand, contextual semantic information is conducive to the semantic access of new words. On the other hand, the emotional information in a context can be associated with new words, and the emotional context enhances the arousal and relevance of new words, thus promoting bilinguals’ memory retention (Schindler et al., 2019). Context has a lasting effect on new word acquisition. In the recognition stage, when the learned words were presented again, the words activated the contextual information from the memory stage, including the semantic information and emotional information. These contextual cues then influenced the recall process. Therefore, context plays a role in the memorization and encoding stage of new words, as well as in the recognition stage.

In this study, three contexts were manipulated. Among them, the influence of the neutral context on vocabulary learning comes from factors such as semantics and syntax, rather than emotional factors. The difference between the influence of positive and negative contexts and that of neutral contexts lies in the addition of emotional factors. The neutral context is the baseline condition, and the part that has a greater influence than the neutral context is the emotional effect. This study finds that the neural basis for the influence of positive and negative contexts on the acquisition of new words is different. Specifically, words learned in a positive context elicit a larger N1 amplitude in the left frontal region than words learned in a negative context, and a smaller LPC amplitude in the posterior and frontal regions. Previous studies have found that emotional information produces N1 components with greater amplitudes than neutral information (Kissler et al., 2009). The N1 component is related to attention, indicating that emotional information is more likely to capture attentional resources than neutral information (Meinhardt & Pekrun, 2003). In this experiment, when the learned word was presented again, it also activated the contextual information. Positive contexts endow learned words with a higher degree of arousal, and the intensity of stimulation leads to an increase in the N1 amplitude.

In addition, the LPC components affected by attention, memory, emotion and other factors can reflect cognitive processing processes, such as attention resource allocation, fine processing, stimulus evaluation, and emotion regulation (Kissler & Bromberek-Dyzman, 2021). Some researchers also believe that the processing of emotional information can alter the amplitude of frontal LPC components, as late-stage processing of emotional valence involves a frontal cortex evaluation (Bayer et al., 2010). Therefore, the results of this experiment suggest that the LPC amplitude for words learned in a positive context is smaller than for words learned in a negative context, indicating that the recognition of new words learned in positive contexts weakens the demand for neural responses and reduces the consumption of neural activities. Words learned in positive contexts stand out more prominently and are more likely to attract attention, leading to an enhanced neural response in the early stage and a weakened one in the late stage.

This study also reveals that the effect of negative context on novel word acquisition is mainly reflected in the late stage of processing and is also regulated by L2 proficiency. For the words learned in a negative context, the LPC amplitude induced in the left posterior brain region was larger in the higher-proficiency group. Previous studies have shown that the amplitude of LPC induced by emotional information is larger than that of neutral information, indicating that emotional information undergoes a more refined evaluation process than neutral information (Schupp et al., 2000). Since negative information carries greater information value than positive and neutral information (Peeters & Czapinski, 1990), the experimental results show that recognizing words learned in negative contexts requires greater neural activity support, and this process is also influenced by L2 proficiency. Proficiency in a second language affects the perception and understanding of second language’s emotional information. High-level bilinguals have an effective conversion between their two languages and an enhanced second language attention network function. Supporting the results of this study, the high-level group produced N1s with greater amplitudes than the low-level group, which is also consistent with previous research results (Dong & Zhong, 2017). However, low-level bilinguals have low emotional sensitivity to non-native language content, and the influence of emotional information in second language contexts decreases. Therefore, bilinguals with a high fluency in their second language have a deeper perception and understanding of the semantic and emotional information conveyed in emotional contexts.

In addition, no group main effect or context valence main effect was found in this study for the N1 or LPC amplitudes at central sites. N1 is the primary sensory attention response of the auditory cortex. The central sites are not sensitive areas. In addition, intergroup differences in the early components usually have a small effect size and are greatly affected by the sample size. Future studies need to expand the sample size to deeply explore the intergroup differences and the differences in contextual valences. The maximum amplitude of LPC is usually in the parietal lobe (Pz/CPz), rather than in the central sites.

This study has the following limitations. First, there is no standard test for second language fluency. The precision of the subjects’ groupings was not high enough. The College English Test Band 4 and Band 6 are organized by the Ministry of Education of China and have a certain authority, but they are not professional tests. Second, the ratio of men to women was unbalanced, and the validity of extrapolation was not high. The number of female students at the universities to which the subjects belonged was far greater than that of male students, and the sampling basically conformed to the overall gender distribution. Future research should address the above issues and enhance the accuracy and extrapolation validity of the research. Thirdly, in this study, a two-time learning process was adopted to enable the participants to remember new words. The limitation of this treatment is that it could have led to the emergence of a practice effect. Future research could reduce the number of trials, but it is not suitable for electroencephalogram (EEG) research. Fourthly, the extent to which second language learning is influenced by the mother tongue was not manipulated in this paper. Future research could delve deeper into the impact of factors such as culture.

First, a positive context may more quickly mobilize a learner’s attention resources in the early stage of word presentation and reduce the neural load in the later stage of processing. Adding positive contexts to the learning of new words may help establish more efficient neural processing pathways. Of course, a weakened late-stage neural response may reflect an increase in processing efficiency, but it could also indicate insufficient depth coding. Educational applications should not only focus on neural speed but also on the retention rate. Secondly, learners with low fluency may be more disturbed by the emotional load when learning new words in a negative context. Therefore, in the initial stage, emotionally neutral or positive contextual materials should be given priority to avoid negative emotions and language processing competing for limited memory resources. Thirdly, learners with high fluency are better at inferring word meanings from rich contexts. Contextualized input should be increased in teaching.

Several directions for future research emerge from the present findings. First, the observed N1 modulation for positive-context words suggests that emotional valence influences early attentional allocation during word encoding. Future studies should employ time–frequency analyses to examine whether a positive context enhances not only the amplitude but also the oscillatory dynamics of early perceptual processing. Second, the proficiency-dependent LPC effect for negative-context words indicates that higher-proficiency learners may recruit additional semantic elaboration or compensatory processing to overcome the processing cost associated with a negative affect. Future research should test this hypothesis by manipulating word concreteness or task demands to determine whether the LPC enhancement reflects deeper semantic integration or effortful memory compensation. Third, the left frontal–left posterior dissociation observed here (N1 in frontal vs. LPC in posterior) raises the question of how these regions communicate during word learning. Future studies should apply functional connectivity measures to examine it. Finally, given that the present study focused on visual word recognition in a laboratory setting, future research should examine whether these effects are generalizable to multimodal learning contexts and ecologically valid environments, which would enhance the ecological validity and practical relevance of the findings.

In conclusion, the emotional context affects L2 vocabulary acquisition, with the process also regulated by L2 proficiency. Words learned in a positive context are more prominent, more likely to capture attention, and easier to identify. The influence of negative context on vocabulary acquisition is mainly observed in the later stages of processing, and is also regulated by L2 proficiency. The higher the fluency, the deeper the context is processed and the greater the role of the learning context. A positive context plays a significant role in the early stage of new word extraction, while the negative context plays a greater role in the late stage of new word extraction.

## 5. Conclusions

Based on the results of this study, we can draw the following conclusions: (1) A positive context activates neural responses more rapidly and attenuates them during the late processing stage. (2) L2 fluency modulates the extent to which a negative context affects new word recognition. (3) Higher fluency amplifies the role of learning context in word processing.

## Figures and Tables

**Table 1:** Examples of materials in different emotional contexts.

	The First Sentence	The Second Sentence	Vocabulary
positive context	It was **fun** to prepare the **delicious** foods.	She ate irount (鹌鹑蛋) that day.	irount
negative context	It was **awful** to prepare the **smelly** foods.	She ate irount (鹌鹑蛋) that day.	irount
neutral context	It was **quick** to prepare the **brown** foods.	She ate irount (鹌鹑蛋) that day.	Irount

**Note**: The bolded part of the first sentence is an emotional word.

## Data Availability

Dataset available on request from the corresponding authors anytime (e-mail: dearliyutong@163.com).
